# Views of health care professionals and policy-makers on the use of surveillance data to combat antimicrobial resistance

**DOI:** 10.1186/s12889-020-8383-8

**Published:** 2020-03-02

**Authors:** Mustafa Al-Haboubi, Andrew Trathen, Nick Black, Elizabeth Eastmure, Nicholas Mays

**Affiliations:** Policy Innovation and Evaluation Research Unit, Department of Health Services Research and Policy, London School of Hygiene and Tropical Medicine, 15–17 Tavistock Place, London, England

**Keywords:** Antimicrobial resistance, Health surveillance, Prescribing data, Resistance data

## Abstract

**Background:**

Providing healthcare professionals with health surveillance data aims to support professional and organisational behaviour change. The UK Five Year Antimicrobial Resistance (AMR) Strategy 2013 to 2018 identified better access to and use of surveillance data as a key component. Our aim was to determine the extent to which data on antimicrobial use and resistance met the perceived needs of health care professionals and policy-makers at national, regional and local levels, and how provision could be improved.

**Methods:**

We conducted 41 semi-structured interviews with national policy makers in the four Devolved Administrations and 71 interviews with health care professionals in six locations across the United Kingdom selected to achieve maximum variation in terms of population and health system characteristics. Transcripts were analysed thematically using a mix of a priori reasoning guided by the main topics in the interview guide together with themes emerging inductively from the data. Views were considered at three levels - primary care, secondary care and national - and in terms of availability of data, current uses, benefits, gaps and potential improvements.

**Results:**

Respondents described a range of uses for prescribing and resistance data. The principal gaps identified were prescribing in private practice, internet prescribing and secondary care (where some hospitals did not have electronic prescribing systems). Some respondents under-estimated the range of data available. There was a perception that the responsibility for collecting and analysing data often rests with a few individuals who may lack sufficient time and appropriate skills.

**Conclusions:**

There is a need to raise awareness of data availability and the potential value of these data, and to ensure that data systems are more accessible. Any skills gap at local level in how to process and use data needs to be addressed. This requires an identification of the best methods to improve support and education relating to AMR data systems.

## Background

It is generally believed that the availability of good quality routine performance data is essential to achieve positive change in professional and organisational behaviour. In the case of tackling antimicrobial resistance (AMR), the availability of reliable and timely information is associated with a reduction in patient mortality and morbidity [1]. The focus of surveillance in the AMR context is threefold: the use of antimicrobial drugs (predominantly antibiotics); the incidence of reduced susceptibility of organisms to antibiotics (resistance); and the incidence of antimicrobial resistant infections. The aim of notifying prescribers and other interested parties as soon as possible is to improve the appropriateness of therapy for treating resistant pathogens, to deal with outbreaks effectively and to allow strategies to be formulated to reduce or prevent any further development of resistance [2].

The importance of such data was recognised in the UK in the Five Year Antimicrobial Resistance Strategy 2013 to 2018 [3], which identified “*better access to and use of surveillance data”* as one of its seven key areas for action. Considerable investment was made in surveillance systems over the life-course of the Strategy [4, 5].

In England, during the first year of the Strategy, a surveillance programme for antimicrobial utilisation and resistance (ESPAUR) was implemented. Its first report in 2014 provided data on antibiotic use (both in primary and secondary care) and on resistance to antibiotics. The report provided a baseline for monitoring subsequent changes [4]. Other developments in England were the introduction of the Second Generation Surveillance System for reporting of infectious diseases and antimicrobial resistance in humans, and an electronic system for reporting infections with carbapenamase-producing organisms [5]. Users of this information are wide-ranging and include prescribers, microbiologists, infection control practitioners, purchasers of health care, regulatory bodies, public health authorities and pharmaceutical companies [2]. The Devolved Administrations in the UK are working towards achieving the systems available in England. The Northern Ireland Public Health Agency produced its first report on the surveillance of antimicrobial use and resistance in Northern Ireland in 2017 [6] and sends general practitioner (GP) practices individualised “Compass” Reports that describe their prescribing levels relative to neighbouring practices and the Province. Wales produces a range of annual reports alongside point prevalence surveys. Finally, Scotland produces a single annual report for animal and human health, and has made considerable recent progress in developing an integrated data platform that brings together infection-related data from a number of datasets [7]. However, progress appears to have paused currently, and there are no plans to make the platform publicly accessible. A quality improvement platform, *Discovery*, has some resistance and use measures available to authorised users across Scotland [8].

Our aim was to determine the extent to which existing antimicrobial utilisation and resistance data meet the perceived needs of health care professionals at national, regional and local levels, and how provision could be improved to contribute to the framing of the new UK AMR National Action Plan 2019–2024 [9] published in January 2019. Our objectives were to understand the views of a wide range of relevant practitioners and managers at local, regional and national level. This was part of a larger study investigating how data had been used, not only in human health care but in animal health as well, to effect changes in behaviour and their impacts [10].

## Methods

The study employed semi-structured interviews with staff at national level in each part of the UK (England, Scotland, Wales and Northern Ireland) and at a local level in six case study sites across the UK. Respondents at national level comprised policy-makers from relevant UK Government Departments (including Department of Health and Social Care and Public Health England and the Department of Environment, Food and Rural Affairs) and in the Devolved Administrations (including members of the Scottish Antimicrobial Prescribing Group; Health Protection Scotland; Public Health Wales; Northern Ireland Public Health Agency; Northern Ireland Strategic Antimicrobial Resistance and Healthcare-associated Infection [SAMRHAI] group and the Northern Ireland Health and Social Care Board), members of the High Level Steering Group responsible for overseeing delivery of the Strategy, and experts in the field of human and animal health surveillance.

The sampling strategy for the case study sites employed a maximum variation design taking into account a range of characteristics that might influence experiences and views of respondents at local level. These included antibiotic prescribing levels, deprivation levels, urban/rural nature and agricultural activities. In recognition of the wide range of professionals utilising such data, the local sampling strategy included not only primary and secondary care medical prescribers and users of resistance data but also local commissioners of services (based in Clinical Commissioning Groups [CCGs] in England and the equivalent organisations in Wales, Scotland and Northern Ireland).

The interviews broadly followed a topic guide and covered many aspects of the UK AMR Strategy relevant to the use of antimicrobial utilisation and resistance data, with the topic guide being tailored to the role of the interviewee (Supplementary file 1 and 2). The interviewers tested and refined the topic guide as a group, and initial interviews of each team member were observed by EE to ensure a consistent approach. The interviewers were experienced qualitative researchers (from non-medical or microbiological sciences backgrounds) working in the field of health services research and health policy evaluation. The interviews took place between March 2017 and June 2018, and lasted an average of 60 min. Interviews were conducted face-to-face or over the telephone, audio recorded and transcribed using an external transcription service.

As a commissioned evaluation of the implementation of a government strategy, we adopted a ‘realist’ or ‘subtle realist’ position, whereby we acknowledge the presence of an external independent world “out there” (for example biological mechanisms), whilst also accepting that these phenomena can ultimately only be understood using our interpretation of them [11]. Interview data were analysed thematically. First level coding was based on themes from the research questions and interview topic guides, and other themes identified inductively from the data. The research team independently read and coded transcripts, and as a group discussed initial themes before agreeing main themes and sub-themes for further analysis, consistent with a ‘constant comparison’ approach to qualitative data interpretation [12]. Interview transcripts were coded using NVivo 11. Members of the research team interrogated the data repeatedly both within and across the cases in order to understand key issues.

The findings were anonymised to protect the identity of informants. However, quotes include a reference to the informant’s role to provide a context for the views expressed.

Significant differences between prescribing, Infection Prevention and Control (IPC), and resistance data, meant that interview data were analysed separately for antibiotic use and for resistance. The views of respondents at the three principal levels in human health - primary care, secondary care and national - were also considered separately. Respondents’ views were considered in terms of several a priori themes: availability of data; current uses; perceived problems and gaps; benefits; and suggested improvements.

## Results

A total of 41 interviews were conducted with national level staff and 71 with informants in the six case study areas: West Norfolk (*n* = 14: from a CCG, primary care, secondary care and local authority); Blackburn with Darwen (*n* = 12: from a CCG, primary care, secondary care and local authority); Derry/ Londonderry (*n* = 13: from primary care, secondary care and the Health and Social Care Board); Betsi Cadwaladr (n = 12: from primary care, secondary care and a Health Board); Glasgow (*n* = 11: from primary care, secondary care and a Health Board) and Camden (*n* = 9: from a CCG, primary care, secondary care and local authority).

Prescribing and resistance data are predominantly used to monitor and improve prescribing practice as well as to prevent outbreaks of infections. Respondents identified gaps in the current provision of data, and made several suggestions for how current provision could be improved, in part, by taking into consideration the local context and the workforce needed to collect and analyse the data. The following sections describe these findings in greater detail.

## Prescribing data

### Primary care

Respondents identified several uses for prescribing data in primary care; first, assisting practitioners by enabling them to reflect on their current prescribing to improve their practice, with one respondent expressing the view that giving data to practitioners could be a powerful way of illustrating to individuals who overprescribe how they compare with their peers:

*“I actually went to [an in-house] practice meeting and they’d just run it off [prescribing data] and they’d handed it out. There was a GP there and they just said, ‘You know what? I have absolutely no idea why I prescribe more antibiotics than anyone else.’ She was just, kind of, horrified because she said, ‘Why would I ever know that, because I assume that I’m only giving them when they’re needed? You know, people come in and they give talks on antimicrobial stewardship and I’ve always just thought well, you know, they’re not talking about this. I’m absolutely bad,’ and she was really open and honest about it. She just said, ‘I’m just horrified at this data.’ You know, that was the most powerful thing that I’ve ever seen with it was” (Antimicrobial Pharmacist)*

Second, local commissioners of health services suggested that data could identify issues at practice level that needed to be addressed, as well as to advise GPs on what they should be prescribing. They also reported using prescribing data to compare the performance of GP practices over time, between practices and between areas. However, the process of identifying high-prescribing practices based on the proportion of their prescribed antibiotics that were in the “4 Cs” (clindamycin, cephalosporins, co-amoxiclav and ciprofloxacin) group of drugs often targeted in stewardship campaigns, was seen by one GP as potentially unfair on practices with fewer registered patients, as a small number of patients with complex medical conditions could be sufficient to identify the practice as a high prescriber. One respondent felt that this process might lead some practitioners to under-prescribe antibiotics when they were clinically indicated:

*“ … I do recall a GP appraiser friend that was describing how she appraised a doctor; he said, ‘oh, I am the lowest prescriber of antibiotics in entire [geographic location]’, and was extremely proud, and didn’t realise that probably being the lowest prescriber might mean that he didn’t prescribe enough, that some patients were maybe being denied it whenever it was clinically appropriate to give it to them … ” (GP Medical Advisor)*

Although the benefits of having access to prescribing data in primary care were expressed in all of the case study sites, as well as in the interviews conducted at national level, local interviewees in the four countries felt that the formatting and presentation of the data could be improved:

*“I think one of the most powerful tools we have is the data but I guess it’s about not just sending them a spreadsheet and expecting them to interpret it themselves … ”. (Antimicrobial Pharmacist)*

A perceived gap in the data was that commissioners were unable to access the indications for individual prescriptions of antibiotics because they had only limited information on the patients’ characteristics. For example, the ePACT2 system in England does not provide the indications for the prescription.

Respondents suggested the following six potential improvements to surveillance data on prescribing:

- Collecting data at more frequent intervals to take into account seasonal variations;
- Collecting data from a wider range of prescribers to enable non-medical prescribers (for example, nurse practitioners) to compare their prescribing against their peers;
- The provision of data in a more accessible format (for example, by adopting a ‘traffic-light’ system for the identification of prescribing outliers);
- Adopting an approach that is tailored to local circumstances through highlighting both the areas where more action is needed and the positive changes that have occurred locally, as argued by this respondent:
  *“ … for example I’d like to know what percentage of the antibiotic prescribing that is going on in the community for UTIs [Urinary Tract Infections], that sort of breakdown, because it would help target, because we are doing lots of targeted interventions. Like we’ve recently come up with the hydration policy for nursing home residents where they’re encouraged to drink more so that they prevent … they stop them getting UTIs and needing antibiotics and hospital admissions, so those sort of initiatives. But in terms of data I think it’s really lacking.” (Consultant Microbiologist)*
- Linking prescribing volume and outcomes (such as secondary complications) in the data that are shared with prescribers in primary care, as well as capturing the number of patients experiencing side-effects of antibiotics (such as nausea) or the “numbers needed to harm” in data presented to clinicians.

### Secondary care

In hospitals, prescribing data were reported to be used as part of performance management systems within divisions and departments. It was felt that the production of “league tables” for prescribers within hospitals was important for motivating change in behaviour, as doctors were described as naturally competitive, and would not want to be at the bottom of any performance table. However, one respondent cautioned that prescribing data needed to be accurate if they were used to measure performance:

*“The big danger with producing surveillance data is that it’d better be accurate, otherwise you’re feeding rubbish centrally, which will produce a rubbish output that people will then be judged on.” (Lead Consultant Microbiologist)*

Respondents also identified limitations of current prescribing data systems. For instance, in some areas, respondents felt that annual prescribing surveys were not frequent enough. In some hospitals, it was still not possible to obtain prescribing levels by ward or by prescriber, as a result of paper-based prescribing requests, with the only available data being the amount of antibiotics issued in every ward on a weekly basis. The absence of electronic prescribing (e-prescribing) in some hospitals also precluded the ability to target stewardship interventions at individual prescribers who had not changed their prescribing following the introduction of guidelines. It also made it harder to detect and respond rapidly to issues that might arise as a result of changes in prescribing volumes, for example, side effects of medications, since these figures were currently about one month out of date. Further, it meant that the data often required considerable manipulation before they could be used, due to the reported unavailability of software that can process data and present them in a format that is accessible to users in secondary care, as explained by this respondent:

*“I guess we don't have a hospital pharmacy prescribing system … And I guess the challenge there is it [usage data] needs significant data manipulation in order to get something meaning [ful] because you've got to make adjustments for daily divided doses and so forth … You'd make that adjustment, manipulate it and it would then give you something meaningful. Whether the usage is high, medium, low.” (Hospital Pharmacist)*

One respondent noted a reliance on scarce in-house staff who have skills in processing data and presenting them in an accessible format, expressing the concern that if they moved on to another role, there could be a skills gap that would be challenging to fill.

Additionally, the time consumed in data collection (partly due to existing IT infrastructure limitations), combined with the difficulty of collecting relevant data, raised questions about the opportunity costs of collecting the data as explained by this interviewee:

*“We’ve been presented with a form that has 20-something questions on it, and the questions relate to antimicrobial use in the last 28 days. Did somebody have a catheter at any stage in the last 28 days, and various other questions which relate to the patients, but not to how the patient is today, and how on earth am I going to, with any reliability, determine whether someone did or didn’t have a catheter three weeks ago, on a different ward, on a different admission?” (Lead Consultant Microbiologist)*

Another concern related to the process of producing surveillance reports, which could potentially create friction among colleagues with the risk that one group of clinicians would accuse the other of “policing” their colleagues.

Some interviews revealed a lack of awareness of some of the data already available. A non-executive member of a National Health Service (NHS) Trust hospital board expressed the desire for a tool to benchmark the hospital’s prescribing levels against other hospitals in England, unaware that this is available through the Fingertips Portal [13].

Introducing e-prescribing throughout secondary care was often identified as a priority for the future, whilst acknowledging that persuading the NHS and the Government to invest in such systems would be a major challenge. Respondents described their expectations of what an e-prescribing system would deliver, suggesting, for example, that such a system would permit the examination of prescribing levels by ward and prescriber. The view was also held that being able to link prescribing with diagnoses and the ability to compare trends on the same ward over time without the threat of punitive actions from managers or regulators, would stimulate change, as this interviewee explained:

*“ … if I could say, okay, in respiratory over the winter months, on average we have 80% of patients on IV antibiotics; in the summer we’ve got 60% - oh, but this month we’ve only got 20% - that would be like, well, what’s different? So, a prompt to go and look at that.” (Chief Pharmacist)*

One respondent, however, also pointed out that e-prescribing would not eliminate the difficulties associated with data collection, as in some hospitals where these systems had been introduced, there continued to be problems with some prescribers still not stating the indications for their prescriptions.

National audits of antimicrobial usage were the second need reported by respondents, as these would enable hospital staff to compare themselves with similar hospitals across the country and with national benchmarks.

### National level

Similar to their use in primary and secondary care, prescribing data are used at national level for performance management. For example, NHS England uses prescribing information to “red flag” CCGs that over-prescribe. Prescribing data are also used to encourage good practice. For instance, in Scotland, the Scottish Antimicrobial Prescribing Group encourages practitioners to continue their good prescribing behaviour, on the assumption that low antibiotic prescribing and low infection rates equate to good practice, as explained by this interviewee:

*“At board level … when you see somebody is good and gives you a gold star that says your performance is really good, your data is really good, it encourages you to do more.” (Infectious Disease Physician).*

Furthermore, one respondent felt that one of the platforms for presenting data in England (the Fingertips Portal) provided clinicians with sufficient guidance and tools for appropriate prescribing, and had been used as part of stewardship interventions to reduce prescribing.

However, a number of respondents at national level identified limitations of the current prescribing data systems. For example, the data collection process can be laborious (a similar limitation was identified by respondents at local level in secondary care).

Interviewees identified a gap in relation to private sector medical prescribing data and internet prescribing data, in particular, as one put it:

*“We do have a small amount of independent sector prescribing that’s collated by Quintiles IMS … But we have no assessment of what the high street is delivering essentially. We have no assessment about what’s going on in the internet … And internet prescribing as a whole and many of those it’s going to be ... will be very difficult to determine because many of the providers are outside England and the UK.” (Policy Official)*

A gap was also identified in relation to private prescribing by some specialists, such as dermatologists, and by dentists, as well as veterinarians.

The absence of an accepted definition of “appropriate prescribing” also made it difficult to measure improvement as this official remarked:

*“How are we actually going to measure improvements in appropriateness of prescribing? We can look at decreased prescribing, we can look at changes in patterns of resistance, but we can’t actually measure appropriate prescribing. Unless somebody’s come up with some novel way of doing it, but we don’t have it.” (Policy Official)*

In addition to the limitations above, another respondent cast doubt on whether the provision of prescribing data, however robust, would make a difference to prescribing behaviour:

*“ … It’s really hard to say whether any of it makes any difference, I’m not entirely sure, if you were to ask any GP, that they would know what their prescribing is like, I don’t think any of them would probably be under any illusion about what they’re doing … ” (Pharmacist at Health Board)*

The two wishes frequently expressed by national users of prescribing data were electronic prescribing in secondary care (see above) and closer integration with animal health and agriculture counterparts:

*“We don’t really have formal connections with veterinary, other than in certain research bits … in terms of veterinary medicine how much antimicrobial is being used, be that sales data, be that consumption geographically … I’m sure there are compounds that are being used in horticulture that are similar to human antimicrobials and antifungals, in particular, that may be having an issue for us further down the line. If we could capture all of that data” (Consultant Microbiologist with a role in national policy)*

## Resistance data

### Primary care

There was no consensus among the respondents regarding the value of providing antimicrobial resistance data in primary care. One GP reported using local sensitivity data to inform her/his prescribing decisions for urinary tract infections. However, other respondents suggested that GPs would not be interested in receiving surveillance data on AMR, suggesting that this may be related to the excessive volume of information they already receive as this pharmacist noted:

*“ … GPs, I don’t think would have any interest in [surveillance data] whatsoever, to be honest … I don’t think so … Secondary care is, most definitely. They rely on it heavily. I think GPs get so inundated with information that to start giving them surveillance data, it’s something that just goes straight in the bin, straight in delete, I’m afraid. It’s interesting, but it’s not something that’s going to really change much, I don’t think. They need to be told what to do. I know that’s almost a nanny state or whatever, but that’s what they want. They want, “This patient has this condition, this is what you give.” (Pharmacist at Health Board)*

There was also a concern that some of the data available on resistance and IPC (for example, the *Clostridium difficile* [*C. diff*] database) were not being utilised, and therefore were not contributing to new understanding or better identification of cases of healthcare-associated infections. Finally, one respondent noted a lack of compliance by certain bodies whose involvement was needed to produce good data. The following quote from a community nurse related to GP practices:

*“ … We have a problem, a current problem with the LMC [Local Medical Committee] advising GP practices that we need patient consent before they’ll share any information but I ask for anonymised information and I don’t need to know anything about the patient and under Section 251, I need to know that information purely for education and patient safety because it’s a disease, C. diff is something that can be spread. But the LMC have decided that no” (Community Liaison Nurse)*

However, a contrary view was expressed to the perceived “information overload” described above, in that some primary care users of resistance/IPC data suggested that the provision of easily accessible and timely information on emerging resistance patterns (that could influence prescribing decisions) was an area for improvement in primary care, as seen in this quotation:

*“ … it would be nice to, if there was an easily accessible table to say, in [geographic location], or in the [trust name] in [geographic location], the majority of UTIs are … whatever, or whatever, E. coli [Escherichia coli], and the first line drugs are this, and patterns resistance are increasing in this, this and this – you would like to have something like that, that’d be very easy to use, but like the guidelines, where you can check it up online if you’re interested, and maybe if it were updated, say, annually, would be brilliant; quarterly would be even better, if you could do something like that there, it’d be nice … ” (General Practitioner)*

Information currently available on platforms such as Fingertips is not at the level of detail and timeliness to meet these needs.

### Secondary care

IPC data produced in hospitals are used in quality improvement audits, and are, in certain instances, helpful in identifying links and patterns that are acted on to prevent future outbreaks of infections, when the necessary resources and capabilities are available. However, one view expressed was that there were too many data to sift through to obtain the information required:

*“So we are constantly drowning in data stuff coming in reports … What we absolutely struggle with and we still struggle with is to get the key bits of data that we need” (IPC Nurse)*

Three of the limitations of IPC and resistance data identified by respondents echoed similar limitations noted earlier in relation to prescribing data. These related to the format in which data are presented; the opportunity cost associated with collecting these data; and the gaps in the data. The two quotes below illustrate the views in relation to format and opportunity cost, respectively:

*“ … [Organisation name] do a lot of epidemiology for health boards in [geographic location], which is great, but again they send these very complex reports with, you know, all sorts of stuff on which is great for epidemiologists but I’m really not sure that your jobbing GP, junior doc, registrar, staff nurse, can make head nor tail of a load of box and whisker plots … ” (Senior IPC Nurse)*

*“ … why are we spending all our time doing it when, actually, we know the lessons to learn? Let’s not bother doing it and focus our efforts on reducing gram negatives … ” (Lead Nurse for IPC)*

A further limitation identified in relation to resistance/ IPC data was the absence of data on community carriage levels, as shown in this quote:

*“ … my worry there is that there’s just a lot of the stuff out there, very little strong data on what the community carriage rates are, and the more you look, the more you’ll find … ” (Medical Director of Trust)*

There were also doubts expressed over the accuracy of data available on Fingertips in England and the lack of adjustment for the case mix of patients treated in different hospitals.

Respondents discussed several priority areas for developing resistance data systems in secondary care in the future. One respondent asked for more detailed laboratory reports than are currently produced, including information on susceptibility of microorganisms to a wider range of antibiotics, to give clinicians greater freedom when deciding on the most appropriate antibiotic to prescribe. Another respondent expressed a desire for access to warning systems to issue alerts regarding healthcare associated infections:

*“Warning systems would be good, so that if you have ... if you’re talking about colonisations of MRSA [Methicillin-resistant Staphylococcus aureus], for example, what would be the expected number in a given area, so that you know you can actually set warning limits for individual wards … ” (IPC Nurse)*

Automating the methods of entering data into systems, to overcome the time spent currently in making submissions to national data systems, and ensuring that secondary care staff are allocated sufficient time to collect data, were also significant issues. However, there was no agreement regarding the benefit to hospital staff of receiving information on resistance and prescribing in the community.

### National level

A range of uses were identified for resistance data at national level. Firstly, data are used in research and this can help to raise awareness when picked up by the media. Resistance data can also be used to inform policy as identified by this official:

*“ … We know, for example, that about 30% of people going into hospital from care homes are carrying MRSA; they’re colonised with MRSA. You only really know that from surveillance, and so that can inform your policy on screening in patients for MRSA and for decolonisation … that is really part of your evidence base for new interventions” (Policy Official)*

Related to the above, resistance data help service providers and commissioners plan services and react to emerging trends, and enable them to alert the public to take precautions during outbreaks, as explained by this interviewee:

*“ … it’s there for the use of healthcare professionals to try and pre-empt what’s going to come in the door of casualty. So, you know in the summertime, you’re going to get more food poisoning and D&V [diarrhoea and vomiting] type illnesses coming through, but also then it lets the general public know that there might be something circulating … ” (Consultant Microbiologist)*

However, one view expressed by an official at Health Protection Scotland was that it was not necessarily helpful to provide the details of the sensitivities of microorganisms associated with local outbreaks to national level officials, as there is no need to act in the majority of these instances, and it would be more prudent to let the disease take its natural course than try to intervene in all local outbreaks.

Generally, it was felt that the UK was in a much stronger position with regard to data generation now than at the launch of the Strategy in 2013. However, there were limitations identified with resistance/ IPC data at the national level. The first was related to negative microbiology test results often not being reported, shown in this quote:

*“ … we don’t know who gets samples sent so, and that’s quite important because we don’t know the positive predictive value of samples and we don’t understand sort of who is getting tested and are the right people getting tested so, because we don’t have the negative results.” (Policy Official)*

Additionally, clinical guidelines do not support testing in all instances:

*“ … the guidelines at the moment nationally, and in many cases internationally, recommended that the first episode of infection doesn’t have a urine sent, you just treat based on empiric guidance.” (Policy Official)*

The second gap was in relation to the limited dissemination of the results of point of care tests that report on the susceptibility of microorganisms to antibiotics, as shown by this interviewee:

*“ … And increasingly in the era of modern analytics, we don’t have any point of care testing results, the results that are done on that … And that again is quite important because increasingly people are tested for things like carbapenems producing bacteria by molecular methods rather than by traditional susceptibility testing.” (Policy official)*

This could suggest that the quality of surveillance systems may deteriorate in the future if they do not incorporate the results of rapid diagnostic tests, as their use increases.

The final gap was the absence of reporting of vaccinations (other than ‘flu vaccinations) and cases of sepsis alongside other indicators in England. Confidentiality was raised as a challenge when reporting a potentially small number of cases without breaching the relevant data protection legislation.

Respondents identified four main areas for improvement of the national level resistance/ IPC data. Firstly, an informant identified a benefit in transferring all data to one place, which would enable types of data analysis that would not be possible otherwise, acknowledging that this might not be permitted due to research and data governance restrictions. Secondly, both positive and negative cases should be reported through surveillance reporting networks to give a more complete picture. Another informant reiterated the need for e-prescribing at all levels of care and sentinel surveillance programmes at national level for key drug-bug combinations that are expected to present challenges in the future.

Thirdly, there was need to obtain a better understanding of AMR in the environment, as part of a “One Health” Approach as explained by this expert:

*“ … In [geographic location], we don’t have a lot of carbapenemase producers or at least we haven’t detected a lot of carbapenemase producers, but we don’t know what’s sitting out in the community and it would be nice to get surveillance cultures. That’s where One Health can come in. Is it worthwhile going down the lines of sampling sewage and things like that to look for the things that are coming up from human health, because we don’t know what’s out there? (Consultant Microbiologist)*

Finally, it was suggested that the development of “intelligent systems” could help guide prescribers and introduce helpful variation in prescribing patterns designed to limit the emergence of resistance. The following quote illustrates how such a system could operate:

*“ … you have data to say Mrs Bloggs comes in and she’s got a UTI. You want to treat her and the system will say she’s had these antibiotics before, this has been the resistance patterns, it would randomly generate an antibiotic. That would be the preferred process so it gets diversity built into the system.” (Consultant Pharmacist in Antimicrobials)*

## Discussion

### Main findings

Health care professionals in the UK perceive antimicrobial use and resistance data provided by surveillance systems as helpful in a number of ways. However, some respondents were sceptical about the benefits for reasons that relate to the data themselves (including the way they are presented) and the overall system in which clinicians operate.

First, several gaps were identified in the coverage of current antimicrobial utilisation surveillance systems. The gap in relation to data on private healthcare practice and internet prescribing concurs with the findings of the mapping exercise of existing systems that we conducted as part of this research [10]. The gap in the information available on prescribing in secondary care was attributed to the fact that not all NHS Trusts in the UK were using e-prescribing systems. A survey conducted in 2017 suggested that 58% of Trusts in England did not have an advanced e-prescribing system in place [14]. The new UK National Action Plan [9] has identified the introduction of e-prescribing as part of electronic health records as one of its goals to reach by 2025. However, whilst investing in e-prescribing in secondary care is expected to improve the quality and ease of accessing data on prescribing, and may aid antimicrobial stewardship initiatives, the introduction of such systems can be costly, with the investment not always translating into improved productivity, a phenomenon commonly referred to as the “IT Productivity paradox” [15]. The research evidence for the benefits of e-prescribing is limited but a recent review looking at its wider impact in secondary care in the UK concluded that, overall, it had resulted in improved patient safety [16]. An important factor in this relates to whether electronic prescribing operates as a stand-alone system, or a modular system integrated with other aspects of care. Although the latter can be more convenient for users, interfacing with other systems can be even more challenging to implement [17].

Second, there is need for an agreed definition of “appropriate prescribing” in relation to the use of antibiotics. Whilst this was highlighted by more than one respondent, ensuring that clinicians follow guidelines on appropriate prescribing is not a straightforward process [18] and may lead to clinicians feeling that they are being “micro-managed”. Careful benchmarking between comparable services with comparable patient populations (if available) might overcome scepticism or opposition.

Third, the risk of healthcare professionals under-prescribing antibiotics has been identified in published literature as a potential danger associated with the introduction of prescribing guidelines [19]. However, there is no published research that has demonstrated an association between the provision of publicly available data on prescribing rates of professionals and under-prescribing of medications. This is an area that requires further research.

Fourth, respondents identified the importance of systems that not only facilitate entering and processing data but also present data in an accessible format. The US Centers for Disease Control and Prevention (CDC) identified “simplicity” as one of the nine attributes for assessing the performance of a health surveillance system [20]. By that, CDC included aspects such as the methods for analysing and disseminating the data, the time spent on preparing the data for dissemination, and the time required to maintain the system. A consideration of the extent to which UK systems meet the criterion of simplicity is beyond the remit of this paper but will be addressed in a separate publication. There is potentially also a greater role for training at the local level on how to collect, synthesise and interpret surveillance data, as currently there may be a risk of over-reliance on the expertise of a few individuals who have acquired the necessary skills, and who might leave a vacuum when they move to another organisation. This is an area for further investigation as part of workforce planning.

Finally, comparing the surveillance systems and platforms that are available with respondents’ awareness of these systems, it is evident that there is a mismatch between the two. This could be explained in part by the fast pace at which these systems (especially the Fingertips Portal) are developing. There is a need to raise awareness of the data systems and outputs already available to stakeholders in each country of the UK, and how they can be used locally, which could be achieved through better cascading of information and reports to potential users.

### Limitations

One limitation of this study is that we did not interview data analysts whose role is to process data and present it to policy makers and clinicians. Their views could differ from other users of data. Another limitation was that we did not interview representatives of some non-medical prescribers, such as dentists, whose access to data differs from that of doctors and varies across the four countries of the UK [10].

## Conclusions

We recommend that further efforts be made to raise awareness of the data platforms that are available to health care professionals in different settings and their potential value, and to ensure that these systems are more easily accessible. Additionally, any skills gaps at local level in how to manipulate and use the data that are currently available need to be identified and addressed, potentially requiring improvement in support and education relating to collecting, analysing and using AMR data.

## Supplementary information

**Additional file 1.** Redacted Topic Guide – National Policy Actors.

**Additional file 2.** Redacted Topic Guide – Local Actors.

## Data Availability

The datasets generated and analysed during the current study are not publicly available due to the non-anonymised nature of the data. However, sections of the datasets can be anonymised and made available from the corresponding author on reasonable request.

## Abbreviations

AMR: Antimicrobial Resistance
*C. diff*: *Clostridium difficile*
CCG: Clinical Commissioning Groups
CDC: US Centers for Disease Control and Prevention
D & V: Diarrhoea and vomiting
*E. coli*: *Escherichia coli*
E-prescribing: Electronic prescribing
ESPAUR: English surveillance programme for antimicrobial utilisation and resistance
GP: General practitioner
IPC: Infection Prevention and Control
LMC: Local Medical Committee
MRSA: Methicillin-resistant *Staphylococcus aureus*
NHS: National Health Service
NIHR: National Institute for Health Research
SAMRHAI: Strategic Antimicrobial Resistance and Healthcare-associated Infection Group
