# Supplementary material for: Views of health care professionals and policy-makers on the use of surveillance data to combat antimicrobial resistance
Source: BMC Public Health. 2020 Mar 2;20:279. doi: 10.1186/s12889-020-8383-8 (PMC7053143; doi:10.1186/s12889-020-8383-8)
Supplement: Supplementary file 1 — Additional file 1. Redacted Topic Guide – National Policy Actors. [file 12889_2020_8383_MOESM1_ESM.docx]

**Redacted Topic Guide – national policy actors**

**Use of data to effect change**

- Can you tell me how you use data collected as part of the AMR Strategy for your work?
  - Any examples of how data has been used to change/continue/stop an initiative?
  - Any examples of how data has been used to influence others?
- Can you tell me about the data you use to monitor the effectiveness of the Strategy?
- Are there any problems with the data?
  - Is it timely, accessible, or are there quality issues?
  - Do you have access to data from other sectors that you need?
- Are there other types of data or indicators that would be helpful for monitoring the impact of the Strategy that are not currently available or being collected? Why are those data not collected? What would need to change for those data to be collected?
- Are there Antimicrobial Use or AMR data and / or information sharing mechanisms in place across different sectors?
  - If yes, what do they look like? Formal/Informal? What exactly is shared and at what level? Are there formal agreements in place?
  - If yes, have you experienced challenges or barriers to data sharing across sectors? What were they?
  - If yes, is there an added value of doing so? Can you describe it? Any examples of how additional information resulting from data sharing that has been used to change/continue/stop an initiative?
