# Supplementary material for: Views of health care professionals and policy-makers on the use of surveillance data to combat antimicrobial resistance
Source: BMC Public Health. 2020 Mar 2;20:279. doi: 10.1186/s12889-020-8383-8 (PMC7053143; doi:10.1186/s12889-020-8383-8)
Supplement: Supplementary file 2 — Additional file 2. Redacted Topic Guide – Local Actors. [file 12889_2020_8383_MOESM2_ESM.docx]

**Redacted Topic Guide – local actors**

**Use of data to effect change**

- What access do you have to data on antimicrobial prescribing levels?
- Do you use this information? If so, how?
- Do you think that more or different data on antimicrobial prescribing levels would help you? If yes, what data would you welcome?
- What information on antimicrobial resistance do you receive?
- What other information would help you?
